# Risk Factors for Fatal Hyperglycaemia Confirmed by Forensic Postmortem Examination - A Nationwide Cohort in Sweden

**DOI:** 10.1371/journal.pone.0164950

**Published:** 2016-10-21

**Authors:** Lotta Walz, Anna K. Jönsson, Brita Zilg, Carl Johan Östgren, Henrik Druid

**Affiliations:** 1 Forensic Medicine Laboratory, Department of Oncology-Pathology, Karolinska Institutet, Stockholm, Sweden; 2 MSD AB, Stockholm, Sweden; 3 Department of Forensic Genetics and Forensic Toxicology, National Board of Forensic Medicine, Linköping, Sweden; 4 Department of Forensic Medicine, National Board of Forensic Medicine, Stockholm, Sweden; 5 Department of Medical and Health Sciences, Linköping University, Linköping, Sweden; Jichi Medical University, JAPAN

## Abstract

**Aims/Hypothesis:**

The aim of this study was to identify risk factors associated with confirmed fatal hyperglycaemia, which could predispose potentially preventable deaths in individuals on glucose lowering drugs.

**Methods:**

A retrospective register-based case-control study conducted on a nationwide cohort with individuals who died due to hyperglycaemia as determined by forensic postmortem examination, in Sweden August 2006 to December 2012. Vitreous glucose was used to diagnose hyperglycaemia postmortem. The forensic findings stored in the National Forensic Medicine Database were linked to nationwide registers. Cases that died due to confirmed hyperglycemia with dispensed glucose lowering drugs were identified and living controls with dispensed glucose lowering drugs were randomly selected in the Swedish prescribed drug register and matched on age and sex. Information on comorbidities, dispensed pharmaceuticals, clinical data and socioeconomic factors were obtained for cases and controls. Adjusted multiple logistic regression models were used to identify risk factors associated with fatal hyperglycaemia.

**Results:**

During the study period 322 individuals, mostly males (79%) with the mean age of 53.9 years (SD.± 14) died due to confirmed hyperglycaemia. Risk factors for fatal hyperglycaemia included; insulin treatment (OR = 4.40; 95%CI,1.96, 9.85), poor glycaemic control (OR = 2.00 95%CI,1.23, 3.27), inadequate refill-adherence before death (OR = 3.87; 95%CI,1.99, 7.53), microvascular disease (OR = 3.26; 95% CI, 1.84, 5.79), psychiatric illness (OR = 2.30; 95% CI,1.32, 4.01), substance abuse (OR = 8.85; 95%CI,2.34, 35.0) and/or living alone (OR = 2.25; 95%CI,1.21, 4.18).

**Conclusions/Interpretation:**

Our results demonstrate the importance of clinical attention to poor glycaemic control in subjects with psychosocial problems since it may indicate serious non-adherence, which consequently could lead to fatal hyperglycaemia.

## Introduction

Diabetes mellitus is a chronic disorder of glucose metabolism causing serious complications, which reduce life expectancy for affected patients [1–3]. At least 10% of global all-cause mortality in the age group 20–79 has been associated with diabetes [4]. The actual number of deaths related to diabetes is however frequently underestimated since individuals with diabetes mellitus usually die due to long-term complications rather than acute events uniquely related to diabetes [4–6]. People with diabetes mellitus are at increased risk of premature death compared with people without diabetes [7–10]. The two main conditions causing death explicitly as a result of impaired glycaemic control are diabetic ketoacidosis and hyperosmolar hyperglycaemic state. Both conditions are consequences of insulin shortage, increased hepatic glucose production and reduced glucose utilisation in peripheral tissues causing the potentially fatal state of hyperglycaemia [11,12].To reduce premature death exclusively related to diabetes, risk factors associated with acute hyperglycaemic events need to be further elucidated. The postmortem diagnosis of acute complications of diabetes mellitus is a challenge due to missing characteristic morphological findings [12–14]. In order to determine death specifically caused by hyperglycaemia, biochemical analyses need to complement the forensic autopsy findings [13]. Vitreous glucose is such a biochemical analysis that has been shown to be a robust method for diagnosing antemortem hyperglycaemia [15]. Worldwide, fatal hyperglycaemia that occurs outside hospitals is probably often overlooked, since collection and analysis of vitreous glucose is not routine clinical practice at every forensic medicine department, and rarely performed by clinical pathologists. During the time of this study vitreous fluid was collected, when available, from all deceased individuals that underwent forensic autopsy and glucose analyses were performed. The aim of this study was to identify risk factors associated with confirmed fatal hyperglycaemia among individuals treated with glucose-lowering drugs (GLD).

## Materials and Methods

### Study design

This study is a retrospective register-based case-control study conducted on a nationwide group of deaths due to hyperglycaemia as determined by forensic postmortem examination in Sweden between August 2006 and December 2012. The cases were identified in the National Forensic Medicine Database (NFMD), which contains exhaustive, structured information about forensic pathology findings and toxicological results of forensic autopsy cases since 1992 [16]. The unique 12-digit personal identification number (PIN) assigned to each Swedish citizen and resident was used to link data in the NFMD with population-based Swedish databases held by the National Board of Health and Welfare, The Swedish National Diabetes Register (NDR) initiated by The Swedish society for Diabetology and the Labor Market Research Database administered by Statistics Sweden. The linkage procedure was performed at the National Board of Health and Welfare, where the PIN was replaced with a serial number to ensure anonymity [17]. Every individual that died due to confirmed hyperglycaemia with dispensed GLD was identified and living controls with dispensed GLD were randomly selected in the in the Swedish prescribed register (SPDR) and matched on age and sex. To minimize the risk of overmatching we did not include additional matching variables [18,19]. Each control was given an index date equal to the date of death of the matched case, all controls were alive at the index date. The study was approved by the Regional Ethics Review Board in Linkoping January 31 2014, Dnr 2014/11-31.

### Forensic Medicine

In Sweden all obvious or suspected unnatural deaths, as well as unexpected deaths, should be reported to the police. The police then has an obligation to request a forensic autopsy with only few exceptions. Relevant information about potential risk factors for fatal hyperglycaemia was collected from the autopsy results, the police reports and medical records when available. Data from postmortem examinations contained detailed information about age, gender, cause of death, manner of death, circumstantial information, medical history including diagnosis, autopsy findings and results of various supplementary investigations such as microscopy and forensic toxicology. For each case, relevant information was retrieved from the NFMD [16]. More detailed information of relevance for the selection and evaluation of the cases were obtained by perusal of the forensic case files. Circumstantial information about identified pharmaceutical drugs at the place of death, suspected substance abuse and if the individual with certainty or probably died alone was retrieved from the police reports. At all forensic autopsies in Sweden, femoral blood, urine and vitreous fluid, when available, are collected at autopsy. All glucose levels in vitreous fluid were determined, by the glucose oxidase /hexokinase method by Hitachi 917 chemistry analyser (Roche, Illinois, USA) [20]. But before that, at one forensic medicine department vitreous samples were also analysed by an in-house blood gas instrument ABL 625 blood gas analyser (Radiometer, Copenhagen), which consistently showed similar results. Routine toxicological analysis as a part of the forensic death investigation included analysis of femoral blood and urine samples with Head Space Gas-Chromatography for volatiles including alcohols and acetone. Screening for pharmaceutical drugs was performed using GC-NPD with neutral and basic extraction as previously described [21]. Increased blood glucose is common in acute illness among both people with and without diabetes. Therefore two independent forensic pathologists evaluated autopsy results, toxicology, police reports and, when available, medical charts to identify cases with confirmed deaths due to hyperglycaemia. The primary inclusion criterion for fatal hyperglycaemia was a vitreous glucose level of > 10 mmol/L and where other causes of death could be ruled out. A vitreous glucose value of 10 mmol/L is equivalent to about 20 mmol/L in blood [22,23]. Further, the vitreous glucose levels will decrease by on average 3 mmol/l the first hours after death, meaning that the included individuals had at least glucose levels of 13 mmol/L in the vitreous, corresponding to approximately 26 mmol/l in the blood, before death [15]. Previous studies have shown that glucose levels >10mmol/L in vitreous fluid correlate significantly to death due to hyperglycaemia [15]. Individuals with high vitreous glucose levels, but who died of other causes, were excluded. Fourteen individuals with glucose levels below 10 mmol/L in vitreous fluid (ranging from 3.5 to 9.9 mmol/L) were included in the study. These individuals had been diagnosed with severe hyperglycaemia and subjected to medical treatment immediately before death. However, actions taken were to no avail, their lives could not be saved and pathologists confirmed death due to hyperglycaemia at postmortem examinations.

### Dispensed Drugs

The Swedish Prescribed Drug Register (SPDR) is a national register held at the National Board of Health and Welfare containing information on all prescribed medicines and pharmaceutical aids dispensed at Swedish pharmacies since June 2005 (100% coverage) [24]. Each pharmaceutical product prescribed and dispensed for personal use is recorded with its name, the anatomical therapeutic chemical code (ATC) date of prescription and dispensed date. Each of the forensic cases or the living controls,18 years of age or older who had at least one dispensed GLD (ATC-code, A10) before death was classified as an adult pharmaceutically treated individual diagnosed with diabetes mellitus. In Sweden, one prescribed drug generally corresponds to a maximum of three months continuous treatment, based on the structure of the Swedish reimbursement system [25,26]. Patients with at least one gap of more than 125 days between dispensed GLD drugs, the year before death/index date, were classified with inadequate refill adherence to GLD [25,27]. Patients with absence of dispensed GLD drugs 125 days or more before death/index date were also classified as individuals with inadequate refill adherence. The SPDR does not include dispensed drugs in hospitals. If individuals were hospitalized during the evaluated period (obtained from HDR), we assumed that GLD were provided by the health care providers during hospitalization. To avoid overestimating refill gaps we subtracted the days of hospitalization from the estimated refill gap when the treatment period overlapped hospitalization.

### Diabetes Related Variables

The NDR has an estimated coverage of more than 90% of all patients over 18 years of age diagnosed with diabetes mellitus in Sweden [28]. For cases and controls variables describing patient characteristics were retrieved from NDR including diabetes duration, type 1 or 2 diabetes, total cholesterol (TC), low-density lipoprotein-cholesterol (LDL-C) levels, blood pressure, body mass index (BMI), smoking habits retrieved as smoking or not smoking, retinopathy, macro albuminuria or renal dysfunction, history of ischemic heart disease or cerebrovascular disease and HbA1c-values. HbA1c analyses were quality assured in Sweden by regular calibration with Mono-S, a high-performance liquid chromatography method until late 2010. In this study, all HbA1c values were converted to NGSP (National Glycohemoglobin Standardization Program) levels (DCCT standard) expressed as a percentage and as a value in mmol/mol IFCC standard unit (International Federation of Clinical Chemistry) in parenthesis. Glycaemic control was estimated by determining Hba1c which asses the average level of blood glucose in the preceding 6–8 weeks. Patients were grouped and finally dichotomized by HbA1c ≥9% (75 mmol/mol) or not. The 9% cut off represents an indicator for ineffective blood-glucose management and an increased risk of hyperglycaemic events based on current literature [25,29,30]. The last registered value in the NDR prior death/index date was used in the analysis.

### Comorbidity and Hospitalization

The national Hospital Discharge Register (HDR) covers information about every hospitalization and outpatient hospital consultation of all Swedish citizens and residents containing hospital admission and discharge with a 100% coverage. For the included patients and controls information on discharge diagnoses, using ICD-10 (International classification of disease) and related health problems were identified from 1997 to the date of death/index date [31]. The discharge ICD-10 codes E10 and E11 indicated diabetes type 1 and type 2 respectively. History of macrovascular events was defined as recorded ischemic heart disease or cerebrovascular disease in the NDR and/or at least one hospital discharge diagnosis of coronary heart disease (ICD-10 codes I21 through I25, I50, and I56) or stroke (ICD-10 codes I60 to I69 and 430 to 438).These diagnoses were retrieved from the HDR before the death/index date. Microvascular complications included recorded retinopathy, macro albuminuria or renal dysfunction (eGFR<60 ml/min/1.73 m^2^) registered in the NDR and/or at least one hospital discharge diagnosis of retinopathy (E10.3, E11.3, E14.3, H360), neuropathy (E10.4, E11.4, E14.4) or nephropathy (N18, N19, E10.2, E11.2, E14.2, Z49, Z992) from the HDR. The ICD-10 code K70 indicated a fatty lever disease. History of psychiatric illness was defined as any discharge diagnosis of ICD-10 codes F10-99 retrieved from the HDR, including depression (F31-F34, F38, F39 and F41) and also mental and behavioural disorders due to psychoactive substance use (F10-19). We classified individuals with substance abuse problems if they had at least one discharge diagnosis of ICD-10 codes F10-19 (indicating substance misuse, including intoxication) [32] and/ or if they had hospitalization or outpatient hospital consultation at a clinic for substance abuse, retrieved from HDR.

### Socioeconomic Factors

For cases and controls socioeconomic factors were collected from Longitudinal integration database for health insurance and labor market studies (LISA) managed by Statistics of Sweden [33]. The database was used to extrapolate relevant information on country of birth, native background (both parents born in Sweden) education level, income level, number of inhabitants in a household, employment status and marital status retrieved the year before death/index date. The unemployment variable was defined as individuals who had no earned income from an employer and/or whose earnings did not exceed the lowest basic amount of the income year retrieved from LISA. The income is based on the annual disposable income of each individual. The individual’s disposable income the year before death/index date was related to all Swedish inhabitants’ disposable income that specific year. We grouped the Swedish population (2006–2012) including cases and controls into quartiles based on disposable income yielding three income categories: low (≤25%), medium and high (≥75%). These groups were created to estimate the possible impact of income on the risk for developing fatal hyperglycaemia. Accordingly educational level was categorized into lower (≤9 years), intermediate (10–12 years upper secondary schooling) and higher (>12 years college/university).

### Statistical methods

Continuous variables with normal distribution (tested with Kolmogorov-Smirnov test) were expressed as mean ± standard deviation, non-normally distributed were expressed as median (25th-75^th^ percentile) and categorical variables were given as number and proportions (%). Differences in baseline characteristics for individuals on GLD compared with individuals with no prescription dispensed of GLD were calculated using Student´s t-test, Mann-Whitney U test or χ2 test. All tests were two tailed and conducted at a significance level of 0.05. This study is one of the first studies investigating factors related to fatal hyperglycaemia with little knowledge from previous research to make more conceptual distinctions. This study is therefore an exploratory study, using logistic regression to identify variables associated with confirmed fatal hyperglycaemia. Variables possible associated with death due to hyperglycaemia among all pharmaceutically treated cases (n = 265) and controls (n = 1325) were dichotomized and odds ratios were at a first step calculated using univariate logistic regression models. Variables showing an association with fatal hyperglycaemia, the dependent variable (p<0.05), were included into multiple logistic regression analyses using a stepwise elimination backward technique, where the least significant variable was removed for each step. We used the stepwise elimination backward technique to improve the prediction power with minimum number of variables. The analyses were performed and the results were expressed as odds ratios (OR) with 95% confidence interval (CI). Complementary analyses were performed where data on cases and controls was stratified according to diagnosis of diabetes (type 1 or 2), recorded in NDR or HDR (cases: type 1, n = 128, type 2, n = 98, diagnosis missing, n = 39). Accordingly, odds ratios for death due to fatal hyperglycaemia were calculated using univariate logistic regression models. Variables showing an association with the dependent variable (type 1 and 2, respectively, p<0.05), were included into multiple logistic regression models using a stepwise elimination backward technique. Data were analysed using SPSS for Windows version 22.0 (SPSS Inc., Chicago, IL, US).

## Results

### Baseline characteristics

**Fig 1:**
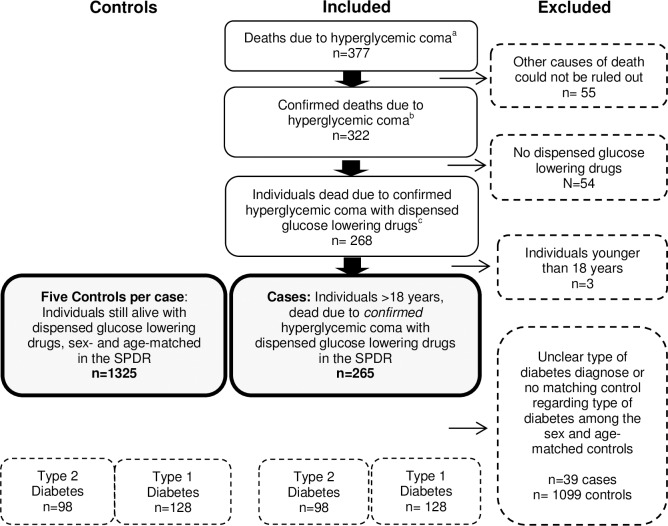
Selection of cases and controls included in the study population. (a) Identified in the National Forensic Medicine Database (NFMD) in Sweden August 2006 to December 2012 (b) Death due to hyperglycemic coma confirmed by two independent forensic pathologists (c) Data retrieved from the Swedish Prescribed Drug Register (SPDR).

From August 2006 through December 2012 a total of 33 447 deaths were subjected to a forensic postmortem examination in Sweden. Of these, 322 individuals died due to confirmed hyperglycaemia. The selection of the study population is shown in Fig 1.

The descriptive characteristics of the individuals with confirmed death due to hyperglycaemia are shown in Table 1. Most of the individuals with confirmed death due to hyperglycaemia were treated with GLD (n = 268, 83%) and almost three quarters (n = 232, 72%) had a history of insulin treatment. A number of individuals (n = 48, 15%) had not been diagnosed with diabetes. The majority of all the forensic cases dead due to fatal hyperglycaemia were males (n = 253, 79%) and most of them (71%) were between 45–75 years of age. The autopsies showed that the vast majority (85%) of all the individuals with confirmed fatal hyperglycaemia had a fatty liver and/or atherosclerosis. Further, 85 individuals (26%) had an on-going infection antemortem. Glucose was detected in vitreous fluid with a mean concentration of 39.5 mmol/L (±19.4mmol/l) in all the cases. The police reports stated that among individuals who died due to fatal hyperglycaemia the majority (n = 243, 76%) were dying alone and almost half of the individuals (47%, n = 151) had no GLD at the scene.

**Table 1:** Characteristics of individuals with confirmed fatal hyperglycaemia, all subjects and stratified on individuals with or without dispensed GLD. Continuous variables with normal distribution (mean ± standard deviation), non-normally distributed (median (25th-75th percentile)).

Variables	All Subjects	Subjects with dispensed GLD	Subjects with no dispensed GLD	
	N(% or SD)	N(% or SD)	N(% or SD)	p-Value
**Number**	322(100)	268(83.2)	54(16.8)	
**Sex (male)**	253(78.6)	210(78.4)	43(79.6)	0.835
**Age (mean)**	53.9(±14.6)	53.2(±14.5)	57.5(±14.3)	0.045
**Min-max**	12–91	12–91	20–89	
**Age group <25**	14(4.3)	13(4.9)	1(1.9)	
** 25–44**	58(18.0)	51(19.0)	7(12.9)	
** 45–64**	180(55.9)	152(56.7)	28(51.9)	
** 65–75**	50(15.5)	36(13.4)	14(25.9)	
** >75**	20(6.2)	16(5.9)	4(7.4)	
**Year of death 2006**	19(5.9)	17(6.3)	2(3.7)	0.895
** 2007**	36(11.1)	30(11.1)	6(11.1)	
** 2008**	46(14.3)	38(14.1)	8(14.8)	
** 2009**	62(19.3)	53(19.8)	9(16.7)	
** 2010**	45(14.0)	39(14.6)	6(11.1)	
** 2011**	54(16.8)	44(16.4)	10(18.5)	
** 2012**	60(18.6)	47(17.5)	13(24.0)	
**BMI kg/m**^**2**^**(median)**	23.1(19.9–26.9)	22.9(19.8–26.6)	223.7(20.8–28.6)	0.089
** <18.5**	32(9.9)	29(10.8)	3(5.5)	
** 18.5–24.9**	175(54.3)	147(54.9)	28(51.9)	
** 25–29.9**	77(23.9)	60(22.4)	17(31.5)	
** >30.0**	35(10.9)	29(10.8)	6(11.1)	
**Atherosclerosis** ^**a**^	273(84.8)	227(84.7)	46(85.2)	0.935
**Fatty liver** ^**b**^	273(84.8)	227(84.7)	46(85.2)	0.935
**Ongoing Infection**	85(26.4)	70(26.1)	15(27.8)	0.880
** Pneumonia**	41(12.7)	35(13.1)	6(11.1)	
** Stomach flu**	13(4.0)	11(4.1)	2(3.7)	
** Pancreatitis**	20(6.2)	16(6.0)	4(7.4)	
** Other infection**	11(3.4)	8(3.0)	3(5.6)	
**Gastric stress ulceration** ^**c**^	105(32.6)	87(32.5)	18(33.35)	
**Abuse**^**d**^**; Alcohol**	130(40.4)	116(43.3)	14(25.9)	0.036
** Other drugs**	36(11.15)	32(11.9)	4(7.4)	
**Vitreous glucose, mmol/L** ^**e**^	39.5(±19.4)	39.6(±19.2)	39.0(±20.4)	0.769
**Min-max**	0.5–110.4	0.5–110.4	9.8–79.9	
** <10 mmol/L**	14(4.35)	12(4.5)	2(3.7)	
** >10 mmol/L**	281(87.3)	232(86.6)	49(90.7)	
**Acetone in femoral blood** ^**f**^	0.33(0.22–0.42)	0.34(0.23–0.43)	0.29(0,19–0,36)	0.046
** <0.1 ‰ acetone**	244(75.8)	209(78.0)	35(64.8)	
** >0.4 ‰ acetone**	74(23.0)	69(25.8)	5(9.3)	
**Dying alone** ^**g**^	243(75.5)	198(73.9)	45(83.3)	0.335
**GLD at the scene**^**h**^	151(46.9)	145(54.1)	1(1.8)	<0.001
**Dispensed GLD** ^**i**^	268(83.2)	268(100)	0(0)	<0.001
** Insulin**	232(72.0)	232(87.5)	0(0)	
** Only Oral GLD**	36(11.2)	36(13.4)	0(0)	
**Known Diabetes** ^**j**^	274(85.1)	268(100)	6(11.1)	<0.001

a. Presence of moderate/severe atherosclerosis in the coronary arteries.

b. Signs and symptoms of cirrhosis and/or liver steatosis.

c. Stress ulceration of the gastric or duodenal mucosa

d. Abuse of psychoactive substances including “misuse, abuse, addiction” regarding one or more drugs

e. Post mortem glucose levels in vitreous fluid

f. Postmortem blood acetone concentrations in femoral blood

g. Alone at time of death stated in the police reports

h. Glucose Lowering Drugs (ATC-code A10) found at the place of death by the police

i. At least one dispensed GLD before death

j. Any dispensed GLD and/or a diagnosis of diabetes mellitus in NDR and/or NPR

### Factors associated with fatal hyperglycaemia

Table 2 shows the predetermined potential risk factors for fatal hyperglycaemia and the results of univariate logistic regression models. Individuals who died due to fatal hyperglycaemia were more likely to have been hospitalized due to psychiatric illness or mental disorders due to substance abuse compared to controls. Living in a single household, a history of substance abuse, low income, lower education and unemployment were social factors that were associated with increased risk of fatal hyperglycaemia. Table 3 displays the results of the multiple logistic regression analyses using a stepwise elimination backward technique. In summary, there were eleven remaining significant risk factors associated with fatal hyperglycaemia. Substance abuse (OR 8.85, 95%CI 2.34, 35.1) and living in a single house hold (OR 2.25, 95%CI 1.21, 4.18) were risk factors associated with fatal hyperglycaemia. Insulin treatment (OR 4.40, 95%CI 1.96, 9.85), inadequate refill of GLD before death/index date (OR 3.87, 95%CI 1.99, 7.53), poor glycaemic control (OR 2.00, 95%CI 1.23, 3.27), a history of psychiatric illness (OR 2.30, 95%CI 1.32, 4.01) or microvascular disease (OR 3.26, 95%CI 1.84, 5.79) were risk factors that at least doubled the odds ratio for fatal hyperglycaemia.

**Table 2:** Results of univariate logistic regressions in cases treated with GLD dead due to confirmed fatal hyperglycaemia versus matched controls. Categorical variables given as number and proportions (%).

**References**	**Cases**	**Controls**		**Valid**		**Crude**	**95%CI**	**p-Value**
**Yes/no**	N = 265	N = 1325		Data		OR	Univariate	
**No = 1.0, yes = OR**	N (%)	N (%)						
**Dispensed drugs (yes/no)**								
Insulin treatment	232(87.5)		781(58,9)		1590	4.90	(3.35–7.17)	<0.001
Refill gap > 125 days GLD ^a^	110(41.5)		382(28.8)		1590	1.75	(1.34–2.30)	<0.001
No refill of GLD before death ^b^	69(25.7)		176(13.3)		1590	2.30	(1.67–3.65)	<0.001
**Diabetes Related Health (yes/no)**								
Type 1 Diabetes, type 2 (reference)	157(61.3)		360(27.7)		1557	4.14	(3,14–5.48)	<0.001
Registration in NDR ^c^	203(76.6)		1214(91.6)		1590	0.30	(0.21–0.42)	<0.001
Current smoker	63(40.9)		196(18.9)		1192	2.98	(2.08–4.26)	<0.001
LDL > 2.5 mmol/L ^d^	71(59.2)		398(52.0)		886	1.34	(0.91–1.77)	0.37
Systolic BP > 140mmHg ^e^	51(28.0)		271(24.0)		1310	1.23	(0.87–1.75)	0.246
eGFR < 60 mL/min/1,73 m^2^ ^f^	20(13.2)		100(10.1)		1146	1.36	(0.81–2.26)	0.25
HbA1c ≥ 75 mmol/mol ^g^ vs. below	85(46.7)		239(20.2)		1367	3.47	(2.51–4.80)	<0.001
HbA1c ≤ 52mmol/mol (reference)	22(12.1)		357(30.1)		1367	**1.0**		
HbA1c 53–72 mmol/mol	71(39.0)		570(48.1)		1367	2,02	(1.23–3.32)	0.005
HbA1c ≥ 73mmol/mol	89(48.9)		258(21.8)		1367	7.01	(4.40–11.18)	<0.001
Diabetes duration > 10 years	127(69.4)		682(57.2)		1375	1.70	(1.21–2.37)	0.002
BMI >30 kg/m^2^	32(21.1)		426(42.3)		1160	0.36	(0.24–0.55)	<0.001
**Comorbidity ^h^**								
Psychiatric illness	139(52.5)		256(19.3)		1590	4.61	(3.50–6.10)	<0.001
Mental disease due to substance abuse	31(11.7)		60(4.5)		1590	2.79	(1.77–4.40)	<0.001
Depression	43(16.2)		91(6.9)		1590	2.63	(1.78–3.87)	<0.001
Drug abuse ^i^	68(25.7)		71(5.4)		1590	6.10	(4.23–8.78)	<0.001
Fatty liver disease	79(29.8)		3(0.2)		1590	187	(58.5–560)	<0.001
Microvascular disease	212(80.0)		575(43.4)		1590	5.22	(3.79–7.19)	<0.001
Macrovascular disease	56(21.1)		226(17.1)		1590	1.30	(0.94–1.80)	0.11
**Socio-economic factors (yes/no) ^j^**								
Native background ^k^	225(84.9)		1051(80.2)		1575	1.39	(0.96–1.99)	0.078
Single household	210(79.2)		520(39.7)		1575	5.80	(4.23–7.96)	<0.001
Married ^l^	22(8,3)		610(46.6)		1575	0.10	(0.07–0.16)	<0.001
Unemployed ^m^	186(70.2)		590(45.0)		1575	2.87	(2.16–3.82)	<0.001
Income in the upper quartile ^n^	36(13.6)		424(32.0)		1590	0.33	(0.23–0.48)	<0.001
>12 years education	77(29.2)		482(37.2)		1561	0.70	(0.52–0.93)	0.014
>12 years education,(reference)	40(15.2)		278(21.4)		1561	**1.0**		
9–12 years, College	133(50.4)		606(46,7)		1561	1.52	(1.04–2.23)	0.030
Maximum 9 years, high school	91(34.5)		413(31.8)		1561	1.53	(1.02–2.29)	0.038

Univariate logistic regression a) p-value from Wald statistics b) classified 89.8%d) Goodness of fit 0.712. Variables showing an association with the dependent variable (cases with fatal hyperglycaemia) (p<0.05) were classified as significant

a. Patients with at least one gap of 125 days or more between dispensed GLD, the year before death/index date.

b. Patients with no dispensed GLD 125 days or more before death/index

c. Patients included in the NDR register

d. Low-density lipoprotein-cholesterol (LDL-C) levels

e. Systolic blood pressure mmHg

f. Estimated glomerular filtration rate (GFR)

g. Glycated haemoglobin (A1c), average plasma glucose concentration, in mmol/mol (IFCC unit)

h. Discharge diagnose codes in HDR

i. ICD codes F10-19 and/ or care taker at a clinic for substance abuse, retrieved from HDR.

j. Socio economic factors retrieved from LISA the year before death / index date

k. Both parents born I Sweden

l. Married or registered partnership vs. reference; unmarried, divorced, widow or widower

m. No gainfully employment, the individual did not receive work or payment from an employer

n. Disposable income yielding three income categories: low (≤25%), medium and high (≥75%)

**Table 3:** Results of multiple logistic regression analyses, odds ratios and 95% confidence intervals for risk factors of fatal hyperglycaemia in individuals treated with GLD.

**N = 265**			
**Variables (yes/no) no = reference**	OR	95%CI	p-Value
**Type 1 diabetes^a^**	**1.93**	1.13–3.30	0.017
**Insulin treatment**	**4.40**	1.96–9.85	<0.001
**No refill of GLD before death^b^**	**3.87**	1.99–7.53	<0.001
**HbA1c ≥ 75 mmol/mol**	**2.00**	1.23–3.27	0.005
**Microvascular disease**	**3.26**	1.84–5.79	<0.001
**Psychiatric illness**	**2.30**	1.32–4.01	0.003
**Mental disease due to substance abuse**	**23.8**	4.57–124	<0.001
**Substance abuse**	**8.85**	2.34–35.1	0.002
**Current smoker**	**1.81**	1.06–3.09	0.029
**Single household**	**2.25**	1.21–4.18	0.010
**Married ^c^**	**0.39**	0.17–0.87	0.022

Variables that showed significance (p<0.05) in the univariate analysis were included in the multivariate analysis. Backward stepwise conditional logistic regression model was applied in 7 steps in all subjects. Hosmer -Lemeshow goodness-of -fit test Chi Square χ^2^ = 4.11; *p* = 0.8

a. Diagnosed with type 1 diabetes type 2 as reference

b. Patients with no dispensed GLD drugs 125 days or more before death/index

c. Married or registered partnership vs. reference; unmarried, divorced, widow or widower

#### Analysis stratified on diagnoses of diabetes, type 1 or type 2

Table 4 shows the results of complementary analyses stratifying the 265 cases on diagnosis of diabetes, type 1 (n = 128) or type 2 (n = 98). Poor glycaemic control, microvascular disease and living in a single household were risk factors that increased the odds ratio for fatal hyperglycaemia and remained valid in all individuals regardless of type 1 or type 2 diabetes.

**Table 4:** Results of multiple logistic regression analyses, odds ratios and 95% confidence intervals for independent risk factors of fatal hyperglycaemia in individuals diagnosed with diabetes type1 or 2 and matched by age and sex.

**4a) Type 1 diabetes; n = 128 cases/controls**			
**Variables (yes/no), no = reference**	**OR**	**95%CI**	**p-Value**
**HbA1c ≥ 75 mmol/mol**	**3.72**	1.37–10.13	0.010
**Psychiatric illness**	**7.50**	2.68–21.04	<0.001
**Microvascular disease**	**4.68**	1.52–14.45	0.007
**Single household**	**10.2**	3.86–27.16	<0.001
**4b) Type 2 diabetes; n = 98 cases/controls**			
**Variables (yes/no), no = reference**	**OR**	**95%CI**	**p-Value**
**Insulin**	**4.03**	0.76–21.3	0.010
**No refill of GLD before death^a^**	**37.5**	2.97–473	0.005
**HbA1c≥ 75 mmol/mol**	**6.02**	1.20–30.1	0.029
**Microvascular disease**	**2.48**	1.38–21.9	0.016
**Current Smoker**	**7.05**	1.64–30.3	0.009
**Single household**	**3.90**	1.34–11.4	0.013
**Married**	**0.09**	0.02–0.46	0.004
**Income in upper quartile**	**0.03**	0.003–0.32	0.001

a. No refill of GLD 125 days or more before death / index date

Variables that showed significance in the univariate analysis were added in to the multivariate analysis. Statistical significance (p<0.05) compared to age and sex matched controls with the same diabetes diagnose. 4a) Backward stepwise conditional logistic regression model was applied in 14 steps in subjects with type 1 diabetes. Hosmer -Lemeshow goodness-of -fit test Chi Square = 5.71; p = 0.57. 4b) Backward stepwise conditional logistic regression model was applied in 11 steps in subjects with type 2 diabetes. Hosmer -Lemeshow goodness-of -fit test Chi Square = 6.71; p = 0.57.

## Discussion

This nationwide study has identified contributing risk factors for fatal hyperglycaemia, by comparing a unique nationwide cohort of individuals with confirmed fatal hyperglycaemia with age and sex matched living controls.

An important finding was that almost 15% of the individuals, who died due to fatal hyperglycaemia, had not been diagnosed with diabetes. Moreover, unidentified diabetes mellitus was confirmed as lack of diabetes related records in the HDR, in the NDR registries, and in the SPDR. In a few publications, DKA death has been associated with undiagnosed diabetes [34,35], although these studies did not include all fatal hyperglycaemic events. Our study included thoroughly investigated individuals where fatal hyperglycaemia was assessed and confirmed by forensic postmortem examination. The majority of these individuals (85%) were older than 45 years of age and probably individuals with undiagnosed type 2 diabetes. One third or more of individuals with type 2 diabetes are undiagnosed at any given time [3,11]. The results presented here confirm that there is a significant amount of individuals with undiagnosed type 2 diabetes and where fatal hyperglycaemia might be the first manifestation of the disease.

We found that the risk of death due to fatal hyperglycaemia was increased among individuals treated with insulin (OR 4.40, 95% CI, 1.96, 9.85). Interestingly, our study also showed that almost half of the cases (47%) had poor glycaemic control at the last health care visit and that their elevated Hba1c values (≥9%) were a strong risk factor for fatal hyperglycaemia, an acute event caused by uncontrolled diabetes. Notable, HbA1c serves as a marker for average blood glucose levels over the previous six to eight weeks before the measurement. Further, our study showed that microvascular disease (OR 3.26, 95% CI, 1.84, 5.79) was a risk factor for fatal hyperglycaemia, indicating that the individuals not only had a single high HBA1c at the last health care visit but probably had a history of persistent poor glycaemic control since microvascular disease is a common consequence of long-lasting uncontrolled diabetes [36]. In addition, our study showed that individuals who died due to hyperglycaemia were more likely to have inadequate refill adherence of GLD before death (OR 3.87, 95% CI,1.99, 7.83), which most likely was a reason for developing hyperglycaemia that contributed to the cause of death. The lack of GLD was recognised by the police, in the majority (>50%) of the cases no GLD were found at the scene. The present study also highlights the importance of psychosocial factors, the risk of fatal hyperglycaemia was doubled in individuals who lived alone or had a history of psychiatric illness. Furthermore substance abuse of such magnitude that it requires healthcare was the most prominent risk factor.

Our findings with several strong risk factors associated with fatal hyperglycaemia complements the results of recent publications in which the risk of all-cause mortality was reported to be increased in patients with diabetes mellitus and a history of poor glycaemic control [8,10]. In Sweden, the National Board of Health and Welfare has issued recommendations with a target level of haemoglobin A1c (HbA1c) less than 7.0% (53 mmol/mol), which is in line with most international guidelines including the American Diabetes Association [37,38]. Our study confirms previous reports that there is a wide discrepancy between recommended HbA1c targets and actual achievements in routine clinical practice, hyperglycaemia defined as HbA1c ≥9% varied between 2.4% up to 17.7% depending on GLD treatment regimen in a resent Swedish observational study [25,39].The total age-adjusted prevalence in Sweden (men and women) was 4.7% in 2012 and the vast majority was diagnosed with type 2 diabetes [40]. The HbA1c values were collected from the NDR which has a high coverage. However, inclusion in the NDR was lower among the forensic cases compared to the controls (77% vs. 92%; p<0.001), which may indicate that the glycaemic control could be even worse among the individuals who died due to hyperglycaemia. Poor glycaemic control is an obvious proof of non-adherence and has been identified as one of the leading causes of hyperglycaemic events [41,42]. Our study demonstrates the importance of clinical attention to poor glycaemic control since it may indicate serious non-adherence, which could lead to fatal hyperglycaemia. According to Medicare & Medicaid services (US) systematic reporting of medication refill adherence from pharmacy databases in real time might be an effective way to alert healthcare providers about alarming refill delay in patients with chronic conditions [43].

Poor adherence to pharmacological treatment regimens in patients with diabetes has been associated with several socioeconomic and psychosocial factors [42,44–49]. The present study extends previous results where psychosocial problems were more closely associated with acute complications than chronic complications in patients with diabetes [11]. In our study patients with no refill of GLD 125 days or more before death was associated with an increased risk for fatal hyperglycaemia end especially in individuals with type 2 diabetes (Table 4). Assessments of living in a single household or substance abuse are not routine in clinical practice but our results indicate that the combination of diabetes and impaired psychosocial status represented by living in a single house hold and substance abuse may be as important contributing factor of fatal hyperglycaemia as other more traditional risk factors.

### Strengths and limitations

The postmortem diagnosis of hyperglycaemia can be a challenge in forensic medicine due to changes in blood and other tissues, but vitreous humour glucose is less affected by postmortem changes due to its isolated position and limited number of cells that may consume glucose after death. The postmortem diagnosis of fatal hyperglycaemia is probably often overlooked, since collection and analysis of vitreous fluid is not routinely performed at all forensic medicine and clinical pathology departments. This study is unique since during the study period, all forensic medicine departments in Sweden have collected vitreous fluid, when available, from all deceased individuals, and glucose analysis were requested upon slightest suspicion of diabetes. The main strength of this study is that all individuals who died due to confirmed hyperglycaemia in Sweden during the study period also were recorded in the NFDM and that the detailed forensic pathology case file was perused in all cases. The PIN makes it possible to link data from NFDM to the nationwide registers to identify associated risk factors for fatal hyperglycaemia. Most deaths in Sweden do not occur at hospitals, but at home, at nursing homes, or at facilities with limited medical surveillance. If an autopsy is requested in such cases it is likely to be a clinical pathology autopsy. We therefore suggest that analysis of vitreous should be performed in the clinical pathology practice whenever an autopsy is performed, since measuring glucose levels in vitreous is crucial for understanding metabolic disorders leading to death and a robust method for diagnosing fatal hyperglycaemia [42,50].

A potential weakness of the study is that we were unable to separate hyperglycaemia with concomitant ketoacidosis from hyperosmolar hyperglycaemia. In 74 individuals elevated acetone values were found in femoral blood. However, blood acetone concentration should not be used in isolation to diagnose deaths due to ketoacidosis [12]. To more safely identify ketoacidosis postmortem, analysis of beta-hydroxybuturate, acetoacetate and acetone should all be analysed [51]. During the time period studied, only few analyses of beta-hydroxybutyrate had been requested. Hence, some cases with no acetone in blood may still have contracted ketoacidosis, and in cases with elevated acetone levels, metabolic derangements due to concomitant alcohol abuse may have contributed to this finding. In this study we have limited our conclusions to observations associated with hyperglycaemia as determined by postmortem vitreous glucose level exceeding 10 mmol/l, regardless of complications. Another limitation is that not all cases of diabetic coma as direct cause of death underwent forensic autopsy. During the study period 477 individuals were reported in the cause of death register [52] 377 (79%) of these underwent forensic autopsy, including vitreous glucose analysis and hyperglycaemia was confirmed in 322 individuals which were included in the present study (Fig 1). The vast majority (80%) of the 100 individuals that never came to a forensic autopsy, but were registered with diabetic coma as cause of death, were hospitalized at the time of death. The cohort that underwent forensic autopsy may not be entirely representative for all individuals who died due to hyperglycaemia and the results should be extrapolated with some caution.

## Conclusion

In conclusion the present study demonstrates the importance of clinical attention to poor glycaemic control in individuals with a history of psychiatric illness, substance abuse and/or living in a single household, since these conditions may indicate serious non-adherence, which could lead to fatal hyperglycaemia. The study adds important knowledge that can be used to identify subgroups that may be at risk for serious hyperglycaemia.
